# A qualitative analysis of collaborative efforts to build a school-based intervention for multiple common adolescent mental health difficulties in India

**DOI:** 10.3389/fpsyt.2022.1038259

**Published:** 2022-11-24

**Authors:** Resham Gellatly, Kendra Knudsen, Maya M. Boustani, Daniel Michelson, Kanika Malik, Sonal Mathur, Pooja Nair, Vikram Patel, Bruce F. Chorpita

**Affiliations:** ^1^Department of Psychiatry, Boston Medical Center, Boston, MA, United States; ^2^Department of Psychology, University of California, Los Angeles, Los Angeles, CA, United States; ^3^Department of Psychology, Loma Linda University, Loma Linda, CA, United States; ^4^School of Psychology, University of Sussex, Brighton, United Kingdom; ^5^Jindal School of Psychology and Counselling, O.P. Jindal Global University, Sonipat, India; ^6^Sangath, Porvorim, Goa, India; ^7^Department of Global Health and Social Medicine, Harvard Medical School, Boston, MA, United States

**Keywords:** treatment development, global mental health, adolescent mental health, implementation science, evidence-based treatment

## Abstract

**Introduction:**

In low- and middle-income countries (LMICs), the gap between need for mental health (MH) treatment and access to services is stark, particularly among children and adolescents. In service of addressing this treatment gap, the current study provides an in-depth illustration of later-stage collaborative design of a school-based, transdiagnostic MH intervention in New Delhi and Goa, India, using a combination of contextual insights from local stakeholders and knowledge derived from the global evidence base.

**Methods:**

Using an inductive-deductive approach to qualitative thematic analysis, we examined coded data from qualitative sources related to experiences of developing and implementing an intervention prototype. These sources included notes from meetings attended by treatment development team members and providers, written feedback on protocol materials (e.g., provider manual, student handouts), field notes reflecting researcher observations, and minutes from weekly clinical supervision meetings.

**Results:**

Results revealed that codes involving cultural/contextual considerations, protocol material and content, and intervention complexity arose consistently throughout treatment development and across document types, illustrating their central role in finalizing protocol design.

**Discussion:**

These findings have implications for the future of mental health treatment development and implementation globally.

## Introduction

Mental disorders are the leading cause of years lived with disability (YLD), accounting for 32.4% of all YLDs worldwide (1). Millions of people suffer from mental illness around the world, and approximately half experience its onset by adolescence (2). Home to nearly 90% of the world’s youth and adolescents (3), low- and middle-income countries (LMICs) are unable to provide even basic care for up to 90% of people with mental health needs (4, 5). This unmet need is often called the global “treatment gap.”

Numerous factors contribute to the global treatment gap, including (in part) scarce and insufficiently trained mental health workers (MHWs) (5, 6). The gap between mental health (MH) service need and treatment access is largest in LMICs, such as India. For example, regarding MHW scarcity, if we conservatively assume 10% of all youth require some type of MH supports, India has one MHW for every 5,520 of these youth, while the US has one for every 37 of these youth (7). Given estimates that hundreds of millions more children and adolescents living in LMICs will experience key risk factors for mental disorders (e.g., child protection risk, inadequate education and employment, substance use) due to the COVID-19 pandemic (8, 9), it is likely that the treatment gap will continue to widen in LMICs (10).

Regarding training, while “clinical psychologist” is defined in India as one with an advanced degree and who meets supervised practice requirements [i.e., see India’s Mental Healthcare Act (11)], the scarcity of such professionals means that non-specialist lay counselors who typically lack this rigorous educational background often must step in to deliver services, with variable outcomes depending on the level of training and support they may obtain (12). The high proportion of adolescents in LMICs such as India, which is home to 20% of the global adolescent population (7), means that the burden of untreated MH disorders in this age group has far-reaching implications for the future of global MH (13).

Researchers have proposed and tested several strategies to address the treatment gap in LMICs, including task sharing [e.g., Lange (14)], adopting or adapting evidence-based treatments (EBTs) [e.g., Murray et al. (15)], and building/assembling new treatments in the local context [e.g., Verdeli (16)]. In recent years, EBTs developed in HICs have been effectively implemented in LMICs (17, 18). These findings offer hope that the treatment gap may be addressed through adopting established treatments. However, implementing an EBT in contexts for which it was not designed has challenges. The majority of EBTs for youth in HICs have been developed in research settings and tested primarily with middle-class, Non-Hispanic White children and families (19), which is not representative of community clinics, let alone LMIC settings.

Given these differences, there is potentially a lack of fit between treatments developed in HICs and the target population in LMICs. Thus, adapting EBTs is seen by some as an essential way to enhance treatment acceptability, feasibility, and effectiveness (20). Typical adaptations include translations, incorporating cultural idioms and/or analogies, reducing the amount of text to increase accessibility for low literacy populations, adding images of local people in materials, and adjusting session length and treatment duration (21). Although several trials have demonstrated the efficacy of EBTs adapted for LMICs (22–26), the adaptation process is extensive, complex, and time- and resource-intensive, and it calls into question whether it is the best solution for scaling up EBTs in LMICs.

Another encouraging solution to the treatment gap involves building a treatment *directly within the planned context* to increase treatment fit with the intended population (27): not starting from scratch, but assembling procedures as building blocks already present within interventions of the evidence base. By testing these interventions within implementation contexts and with direct input from local treatment providers and participants (27, 28), this approach capitalizes on user-centered product development to increase acceptability and usability, raising the likelihood for sustainability (29).

In addition to considering the target population’s unique needs and preferences, building treatments in context considers setting-specific constraints during the design process. This approach acknowledges that the context of care in community clinics, especially those in LMICs, bears little resemblance to clinical trials in HICs and thus further complicates implementation. Compared with HICs, mental health providers in LMICs are typically non-specialist lay counselors who have limited training, limited resources (e.g., supplementary materials, printers, therapy rooms) and lack infrastructure (e.g., access to mental health professionals, clinics, coordinated care).

The mission to build a treatment that fits with real-world contexts has led to innovations in MH treatment design, culminating in the creation of systems that help providers address the complexities of working with youth in diverse, resource-limited settings. For instance, there is compelling evidence that Managing and Adapting Practice [MAP, Chorpita and Daleiden (30)] can be used to build effective and acceptable, individualized treatments [see Chorpita and Daleiden (31)] by supporting providers in applying evidence-based elements and systems of coordination. For example, to integrate flexibility and case-specific tailoring within its structured protocol, MAP leverages both clinical procedures that the design team determined in advance (“design-time”) and decisions that the providers can implement in the moment (“run-time”). PREMIUM, a Program for Effective Mental Health Interventions in Under-resourced Health Systems, is another design-in-context methodology that was used to build two lay-counselor-delivered MH treatments in India, which outperformed usual care and were found to be cost effective and acceptable (32–34). Given the success of these approaches, the PRIDE initiative investigated whether a similar framework could be used to develop a scalable treatment for adolescents in India. Development of the first step of the stepped care architecture (35) and the second step (36) have previously been described in detail, as have the findings from an acceptability and feasibility pilot of Step 2 (37). This current study adds to this body of work and brings a unique perspective by offering a qualitative look at challenges and successes that arose throughout the development process in the words of multiple stakeholders involved in intervention design and testing. Findings have implications for treatment developers designing for populations with complex needs in resource-limited contexts.

## Materials and methods

### Background of the PRemIum for aDolEscents study

PRIDE (PRemIum for aDolEscents) is a research program that aims to (1) develop a transdiagnostic, stepped-care intervention targeting common mental disorders in school-going adolescents in India, and (2) evaluate its effectiveness. PRIDE is composed of two sequential treatments of incremental intensity (Steps 1 & 2), so that adolescents who do not benefit from Step 1 receive Step 2. This stepped care approach increases accessibility and efficiency (38), which is particularly important in low-resource settings. In the PRIDE intervention, Step 1 is a brief (4-5 session), low-intensity problem solving intervention guided by lay providers and supplemented by a printed comic with psychoeducational stories and self-completed practice exercises (35). Step 2 (36) is a high-intensity, face-to-face intervention originally intended to be delivered by qualified psychologists. Later Step 2 piloting supported a single-provider model in which lay counselors delivered both intervention steps.

### The present study

Considering adolescents’ heightened MH risks and need for accessible EBTs in LMICs, our treatment development team was tasked with using the MAP system to design Step 2, a modular, multi-problem psychological treatment in low-income schools in India for adolescents who did not respond to the first level of treatment in the stepped care model. The treatment development process was composed of three formative phases: (1) *context review*, (2) *adopt-adapt-assemble*, and (3) *design and build* (see below; 36). The design process was guided by a model of treatment integrity (39). This framework prioritized establishing expected outcomes in four domains (resources, e.g., treatment materials, participants, space, time; activities, e.g., specific practices, service encounters; coordination, e.g., fit, flow; and outcomes, e.g., symptom reduction, functional improvement) and designing the intervention to meet these targets given the setting constraints.

### Context review

In order to identify the setting constraints and population needs to maximize fit, an intensive *context review* was conducted by members of the Intervention Working Group (IWG), comprised of an intervention development team at UCLA who worked closely with research coordinators and clinical experts based in the UK and at Sangath, India, and the Scientific Advisory Group (SAG), a group of international researchers and clinicians with expertise in global mental health and treatment design. This phase included in-person visits to schools in Goa and Delhi, India, and review of the literature, local policies, and research conducted by various Sangath teams to assess student and provider needs and preferences. The context review resulted in a *Statement of Values and Preferences* that informed the team’s design decisions in the next phase of development [see Chorpita et al. (36)].

### Adopt-adapt-assemble

The second phase of formative activities, *adopt-adapt-assemble*, centered around deciding on a treatment design strategy that would result in a protocol that satisfied the values and preferences identified in the context review phase. The team considered whether it would be appropriate to *adopt* an existing EBT and transport it to the PRIDE context as-is; *adapt* a candidate program by modifying certain features to increase fit with the target context; or *assemble* the treatment specifically for the context using evidence-based practices and strategies. The IWG and SAG spent a significant amount of time discussing these potential pathways before deciding that assembling the protocol for the context was the best option given the distinct features of the setting and the values and preferences identified in the prior phase of activity, such as the need to balance flexibility and structure within a relatively complex, multi-problem protocol delivered by a non-specialist workforce. This second phase of formative activities resulted in the *Parameter Specification* output, which mapped onto the dimensions specified in the *Statement of Values and Preferences* and was used to organize development of the protocol blueprint in the third and final phase of formative activities: *design and build*.

### Design and build

The *design and build* phase began with identifying and selecting practices to include in the Step 2 protocol. Candidate practices were identified by reviewing the MH literature with the Distillation and Matching Model [DMM, Chorpita et al. (40)], matched with a set of common MH problems identified in the local adolescent population, and selected through consultation with local experts [see Boustani et al. (41), for a detailed account of this process]. According to the literature, the problem types most reported by the focal age group (12 to 17 years) corresponded to ten relevant practices (e.g., rapport building, exposure, problem solving). A treatment development team at UCLA worked closely with research coordinators and clinical experts based in the UK and at Sangath, India, to form the IWG, which discussed the practices’ suitability for the local context: the acceptability of practices for the adolescent population and the feasibility of a non-specialist workforce to deliver the practices. The current study describes the process of building the Step 2 treatment from post-practice selection through the conclusion of two clinical case series in Goa and Delhi. This study’s primary objective was to qualitatively analyze how large, cross-national teams utilized both local knowledge and the broader research evidence base to collaboratively navigate intervention design for multiple MH problems.

### Design preferences

Based on the literature and user-reported experiences with the Step 1 protocol (35), we identified design preferences for the Step 2 protocol. These preferences fell into two categories: (1) content (i.e., characteristics that met the needs of the target population and providers), and (2) format (i.e., aspirations for the protocol’s look and feel.) By building clinical procedures that could be determined in advance by the design team (“design-time”) as well as those that the providers could make in the moment decisions (“run-time”), we further desired to facilitate flexibility and case-specific tailoring within the structured protocol.

#### Content design preferences

Content design preferences concerned the intervention’s data and logic components, which were largely determined in earlier phases of treatment development. For example, the intervention developers and IWG researchers identified what practices were indicated given the data on MH concerns in the target population, how those practices fit together, who should receive what practices based on initial assessment and ongoing monitoring scores, and how to support providers in treatment delivery. Major content design preferences included: (a) multi-problem focus; (b) efficient to learn and deliver; (c) feasible within a limited (∼35 min) clinical encounter; (d) appropriate for a workforce with varied educational backgrounds; (e) able to be delivered by providers after a condensed training; (f) able to be delivered within a set number of sessions for a problem; (g) fit within PRIDE’s stepped care model.

#### Format design preferences

Format design preferences were articulated to design a protocol interface that met its end users’ wants and needs. These preferences were specified as: (a) culturally appropriate for India; (b) intuitive and suitable for the workforce; (c) engaging for youth; (d) accessible for youth with varying levels of literacy; (e) minimizing barriers to implementation (e.g., reducing the number of assessments, providing worksheets rather than workbooks).

### Participants

#### Intervention working group

The IWG communicated frequently by email and in meetings that were held at least every month and sometimes as often as weekly. The IWG members who most regularly participated in meetings included the PI on the project (VP), the treatment development team from UCLA (BC, MB, RG, KK), and members of treatment development team based in India (KM) and the UK (DM).

#### Provider sample

This study included MH providers (*N* = 5) employed by Sangath, a non-governmental, non-profit organization conducting research and providing psychosocial services across India. All five providers and the first author of this paper (RG) participated in weekly group supervision meetings. One provider (KM) was part of the IWG and joined IWG meetings regularly throughout the pre-implementation and implementation phases. Other providers joined IWG meetings less frequently. Participants consisted of two post-doctorate clinical psychologists, two master’s psychologists, and one expert provider (i.e., no degree in psychology but significant experience as a provider on mental health treatment studies). All providers were Indian nationals and self-identified as female. The average age of providers was 30.8 years (*SD* = 4.55, Range = 26-38) and the mean number of years of experience in mental health services was 7.8 (*SD* = 2.39, Range = 5-11). Four providers had previously delivered services in secondary schools (the equivalent of ninth and tenth grade in the Indian school system); three had been therapists on research studies or in community mental health settings; and all had experience working in clinic and hospital settings. Providers reported delivering services in English and Hindi; one provider also provided treatment in Konkani and Marathi.

### Data sources

To describe the process of developing a stakeholder-informed MH intervention for adolescents in a low-resource setting, we coded qualitative data sources, including meeting notes and written feedback on protocol components from the pre-implementation phase (N = 55), meeting and field notes from the implementation phase (N = 22), and supervision notes (N = 23) from the implementation phase. Data were collected between November 2016 and May 2019. All study procedures were approved by the Institutional Review Boards at the University of California, Los Angeles, Harvard Medical School, Sangath, and the Indian Council of Medical Research.

#### Meeting notes

Meetings primarily included members of the IWG, although providers joined meetings periodically. Detailed notes were taken by the first author (RG) at meetings conducted over video conferencing beginning in November 2016, after the intervention practices had been selected and finalized. Notes were circulated via email to the broader research team, including the PI (VP) of the study, who then reviewed the suggested protocol modifications and made recommendations.

#### Written feedback on protocol materials

Throughout the iterative revision process, members of the IWG and treatment providers drafted the protocol and provided in-text feedback on the protocol. Edits and comments relevant to protocol development were extracted from protocol materials and uploaded for coding.

#### Field notes

Field notes are a critical component of qualitative research, providing rich contextual information to inform data analysis (42–44). The first author (RG) took field notes primarily to document informal conversations related to treatment development and implementation that occurred amongst the IWG and providers. For example, a running document of concerns and ideas raised by providers outside of scheduled meetings was maintained to capture real-time feedback, which was later given to the larger team.

#### Supervision notes

The clinical case series took place from August 2018 through May 2019. Thirty-two total students entered Step 2, half from Goa and half from New Delhi. Fourteen students completed treatment in Goa, and two students completed treatment in New Delhi. Providers participated in weekly peer supervision meetings to discuss cases and challenges with treatment delivery. Throughout the clinical case series, different providers took turns taking supervision notes, which were later coded. To ensure accuracy of note content, the first author (RG) cross-checked a random sample of notes against their corresponding audio recordings.

### Data analysis

All the above data sources were coded using Dedoose, a qualitative data analytic software program. A primarily deductive approach was used to generate *a priori* codes and definitions from (1) initial intervention design values and preferences, and (2) specific design parameters generated in earlier design phases (36). This framework was applied to a subset of “gold standard” documents, randomly selected from each category of document type at each study phase, that the master coder first coded and the coding team subsequently further refined using the qualitative analytic method of coding consensus, co-occurrence, and comparison (45, 46). The remaining documents were divided among the coding team for independent coding using the same qualitative analytic method.

The coding team included eight undergraduate research assistants who learned coding theory and procedures via 2 h long training sessions. These included a group discussion of the coding scheme, the Dedoose coding process, current codes with examples, and potential new codes. Discrepancies that arose during practice coding were resolved as a group. Next, the master coder reviewed all remaining coding, provided written feedback, and discussed questions. Final codes were applied, defined, and refined through consensus. Thus, emergent codes were inductively derived and organized into thematic categories.

Coded documents were categorized into two phases of the clinical case series and analyzed separately. These phases included: (1) *pre-implementation* (November 2016 through July 2018) and (2) *implementation* (August 2018 through May 2019). Pre-implementation documents were further divided into two categories: (1) supervision notes with providers only, and (2) field notes and meeting notes with the IWG, which providers sometimes joined. These categories were also analyzed separately to examine differences in the type and frequency of applied codes. When code categories included both primary (parent) codes and secondary (child) codes, only secondary codes were included in the analysis to specify code application.

## Results

The following results highlight codes applied in 55% or more of documents in at least two of the three categories examined. Please see Table 1 for definitions, exemplar quotes, and frequencies of all codes.

**TABLE 1:** Presence of codes applied to notes in pre-implementation phase and implementation phase.

			Frequency of code application (%)		
			Pre-implementation	Implementation	
Code	Definition	Exemplar quotes	Meeting notes (*N* = 55)	Meeting notes and field notes (*N* = 22)	Supervision notes (*N* = 23)
Assessment	Discussions revolving around what measures to include in the initial and outcome assessment battery (e.g., RCADS, YTP, SDQ) in order to assess a) eligibility, and b) clinical outcomes at the end of treatment	“Right now, students are completing the YTP, SDQ, and emoji mood rating regularly, along with homework that often involves them taking mood ratings. The RCADS is completed with the researcher at baseline. The amount of time and paperwork required to complete these [assessment] forms is a burden to the student and the therapist.”	40.0	72.7	21.7
Clinical decision making	Discussions involving clinical decision making: how to guide providers with limited clinical experience to make decisions about which problem to target and which module to choose for that problem, as well as decisions about transitioning between modules, repeating content, and ending treatment	“What variables do we need to look at to better understand which [treatment] flow to take?”	49.1	86.4	78.3
Complexity – Provider	Discussions about how to balance complexity of the intervention with making it simple enough to be used by non-specialist providers, thus making it scalable (i.e., able to be delivered by a large non-specialist workforce), while not compromising features that can be used by providers with increasing experience.	“.give the beginner therapist more structure but as they become experienced, they can have more flexibility.”	50.9	63.6	73.9
Complexity – Student	Discussions about making Step 2 content and material appropriately complex for student participants.	“Have something visual/graphic that is interactive; eliminate writing as much as possible.”	61.8	68.2	95.7
Cultural and contextual considerations	Discussions involving how to take culture and context into account when developing Step 2	“Use visual aids that are culturally appropriate/relevant to Indian youth: plate of rice instead of fork and knife; inclusion of emotion scale instead of mood thermometer; use of X out of 100 rather than percentage”	83.6	77.3	69.6
Demand for services	Discussions about how to meet demand for services (e.g., school-wide sensitizations, other ideas for Step 0). May also involve discussions about referral numbers.	“Update on referral numbers for Step 1 and implications for Step 2. Team is working on ways to increase referrals in Goa”	40.0	27.3	8.7
Eligible participants	Discussions about eligible youth for consent, measurement, and treatment	“Determine whether case is severe enough to warrant treatment (SDQ) and then focus (Top Problems)”	0.0	0.0	0.0
Engagement	Discussions about engagement, i.e., how to keep student participants engaged in treatment and prevent dropout.	“Use engagement strategies available from the literature and include in workbook, e.g., instilling optimism that the program works.”	60	59.1	95.7
Level of care	Discussions on level of care (i.e., Step 1, Step 2, referring out); how to determine whether a youth participant goes into Step 1, Step 2, or is referred out; discussions about how to transition between steps (i.e., level of care)	“Since many students in Delhi won’t have gone through Step 1, we may want to spend more time thinking about how to engage them and discuss psychoeducation more.”	69.1	77.3	60.9
Funding	Discussions involving how to consider the eventual transition of the study into a fully integrated program within schools	“In the spirit of sustainability planning, we need to keep in mind that we don’t want to burden schools by having them administer and score measures in a timely way and then triage to a certain level of care; easier for everyone to enter the lowest level and then non-responders step up.”	12.7	4.5	0.0
Guardian involvement	Discussions about including guardians (i.e., parents, caregivers) in treatment	“Adolescent specific content is often family focused. How involved are parents going to be?”	29.1	22.7	13.0
Impact of school calendar	Discussions involving the impact of the school calendar (e.g., exam periods, holidays) on treatment delivery	“Would be a good idea to do the psychoed and engagement sessions close together – might increase engagement; then spacing in behavioral to allow for practice; then could space out near the end, but we had to try to fit in sessions whenever we could because of the breaks for exams and holidays.”	21.8	40.9	43.5
Mode of delivery	Discussions about the ideal mode of delivery for Step 2 (in-person, telephone sessions during breaks)	“Students don’t have much experience with smartphones; parents and peers would wonder why they got it; teachers raised concerns about smartphones/tablets being stolen or damaged.”	38.2	40.9	4.3
Monitoring	Discussions revolving around what measures to administer as ongoing symptom monitoring during Step 2 treatment (e.g., YTP, SxS, mood rating with smiley face); youth and provider report of emotional, behavioral, and risk status across sessions	“Consensus from providers is that the YTP is most useful, takes less than 5 min. SxS is difficult for the student to read, they don’t reflectively think about responses.”	14.5	63.6	56.5
Practice content	Discussion related to development of practice content), including conversations about prioritizing concrete behavioral over abstract techniques	“Embed some cognitive work in the beginning of the behavioral modules to help students understand the rationale behind learning these skills”	70.9	81.8	82.6
Privacy	Discussions about how to define and convey privacy and confidentiality safeguards in sensitization, consent, and treatment activities	“Confidentiality concerns: youth do not want to take it home and do not want to be seen with it at school (stigma?). Fear that friends or siblings will see it and read it.”	7.3	0.0	26.1
Provider experience	Discussions about providers and experts, as well as providers’ level of experience more broadly, including training and education	“In Delhi, Step 2 will be delivered by psychologists per government requirements. In Goa, delivered by psychologists and counselors.”	45.5	59.1	21.7
Provider-facing materials	Discussions on developing provider-facing materials: manual, flipbook, clinical record form, appendices	“The manual may need to have guideline for providers covering three scenarios that are possible in terms of time available for delivery.”	69.1	90	95.7
Quality assurance and improvement	Discussions about training, supervision, and expert consultation.	“Continue developing a supervision model for peer supervision that serves important formative and normative functions, as well as restorative functions related to preventing burnout, boosting team morale, normalizing difficult cases, etc.”	45.5	77.3	47.8
Session duration	Discussions involving the duration (number of minutes) of a session.	“The session duration should fit within classroom period. Given our context, it will be difficult for student to miss more than one period.”	20.0	27.3	52.2
Space	Discussions involving how to maximize privacy within semi-private space (e.g., curtain, positioning of seating, rooms)	“Step 2 has a lot of material – able to find place for it in the clinic but need to think about where to store everything in schools; hard to find place/carry things”	7.3	4.5	26.1
Spacing of sessions	Discussions involving the optimal spacing of sessions (semiweekly to weekly sessions)	“Sessions can be spaced so that initial sessions take place twice a week and gradually on a weekly basis, as this allows adolescents more time for practicing what they have learnt.”	10.9	45.5	13.0
Step 2 Practices	Discussions involving what practices (e.g., relaxation, behavioral activation, assertiveness and communication, exposure, problem solving, cognitive) to include in the protocol	“Substance abuse is rarely a concern except for chewing tobacco. Current measures are not picking up on trauma although it is possible that the kids have experienced trauma.”	41.8	54.5	21.7
Student-facing materials	Discussions on developing youth-facing materials: consent forms, youth measures, handouts, Youth Top Problems dashboard, and flipbook	“Experience has been positive with visuals, helps to stimulate conversation and is a good memory aid. Interactive, not so boring. It feels nice for the kids. Key messages to have in pictorial representation – easy to follow and remember.”	80	59.1	43.5
Student-provider relationship	Discussions on the nature of the student-provider relationship and how to balance a collaborative style with representation of provider as an “expert” due to cultural role expectations	“Students are both happy and sad to end; you are spending more time with them, and you are more familiar. One student wanted my number to be able to call for help in the future.”	29.1	45.5	78.3
Targets	Discussions about using a transdiagnostic/multi-problem approach for the three targets of mood, anxiety, and conduct, especially at lower steps (e.g., general cognitive-behavioral skills, relaxation)	“Consider 3 ‘core’ skills that everybody gets: problem-solving, communication skills, relaxation skills for all 3 flows and then adding practices according to primary diagnosis: exposure, cognitive? Would make it easier for counselors to learn… provide examples relevant to each flow to maximize utility.”	34.5	50.0	13.0
Theory	Discussions regarding the theoretical framework behind both steps (i.e., Step 1 as a problem-focused coping intervention and Step 2 as primarily emotion-focused coping while building on problem solving skills)	“Think about theory of stress-coping model; Step 1 is primarily about the problem/problem solving. Then there is emotion-focused coping and appraisal of the stressor *and* one’s own coping ability (efficacy).”	25.5	45.5	17.4
Treatment architecture	Discussion about treatment architecture (e.g., order of modules, number of sessions per module, which modules should be optional, optimal dosing)	“Currently, the flow seems rigid; if it has a more specific focus on integrating cognitive and behavioral, will be easier to go between these concepts.”	50.9	72.7	47.8
Treatment duration	Discussions involving the duration of treatment episode (6-10 weeks)	“The current Step Two seems difficult to scale with the time it requires to deliver the intervention with multiple modules. Such interventions are difficult to deliver in school context and may not be acceptable by School authorities. What we need to look for a most simple, short and scalable interventions.”	20.0	59.1	26.1

### Continuity across development

Six codes arose consistently throughout pre-implementation and implementation and across document types: *Provider-Facing Materials, Cultural/Contextual Considerations, Engagement, Level of Care, Practice Content*, and *Student Complexity*. The codes for *Provider Complexity*, *Clinical Decision Making*, and *Monitoring* were not frequently applied during the pre-implementation phase; however, they were coded at similar rates in the meeting notes, field notes, and supervision notes during the implementation phase. *Student-Facing Materials* arose in two categories (i.e., meeting notes during the pre-implementation and implementation phases) but was applied less frequently in supervision meeting notes at implementation.

#### Provider-facing materials

The *Provider-Facing Materials* code captured discussions on developing provider-facing materials, which included a manual, flipbook, clinical record form, and appendices. It was the most frequently applied code (84.9%) across document types. It was most prominent in the implementation phase (i.e., 95.7% in supervision sessions, 90% in meeting and field notes) compared with the pre-implementation phase (69.1% in meeting notes).

Discussions about provider-facing materials largely related to the question of how to design dynamic materials that met providers at their varying levels of experience. The team aimed to balance structure and flexibility by developing materials that would grow with providers as they gained experience with the protocol. With this aim in mind, the manual was conceptualized as a comprehensive support that providers would likely rely on with their first cases. The flipbook was designed as a pared down version of the manual or “quick guide” that providers might reference instead of the more detailed manual once they gained experience with the treatment. At pre-implementation meetings, members of the team recognized a need for more support in the manual, suggesting various “adaptations for counselors” such as “being more directive and thorough in the manual” by “direct[ing] counselors to ask specific questions” after completing each section (e.g., “Ask the student if they have understood”). This suggestion and others were offered by providers, and the design team incorporated their feedback into the many protocol iterations that providers reviewed. However, some issues arose only after the manual was piloted in the implementation phase. For example, in supervision it was noted that “there are a lot of loopholes in the manual, like when to use the treatment planner,” and a suggestion was made: “Could put instructions for how to use it in the manual, like bringing it out at the start of each module to orient the student to where they are in the treatment.” The design team was able to make these changes to the manual in real time to give providers the support they needed. The prevalence of this code throughout development highlighted the importance of involving providers, as end users of these materials, throughout the design process, and actively eliciting feedback to identify and address concerns with expedience.

#### Cultural/Contextual considerations

The *Cultural/Contextual Considerations* code captured discussions involving how to take culture and context into account when developing Step 2. It was frequently applied (76.8%) across document types, particularly in the meeting notes of the pre-implementation phase (83.6%). Conversations about culture were critical, as team members recognized the importance of designing a protocol that would be culturally acceptable to provider and student participants, as well as feasible to deliver given the resource-constrained school context. Culture-related issues were discussed frequently during pre-implementation, whereas context-related issues were more common during implementation. Indian team members were often in the position of explaining cultural norms to the U.S.-based team, who took the lead in drafting the initial protocol materials. For example, in the pre-implementation meeting notes, Indian team members recommended the “use of local terms for anxiety/depression (‘tension’)” and “visual aids that are culturally appropriate/relevant to Indian youth.” For the visual aids, decisions ranged from how to present food to how to depict mood ratings and their corresponding scales and what ranges of skin colors to illustrate. Indian team members’ feedback was essential to ensuring that the resulting protocol was a strong fit with the culture. Similarly, in informal discussions, development team members noted the need to “consult with young people [the students]” when selecting images and content for practice activities [e.g., “How did we choose the four images for activities in BA (behavioral activation)? Did we consult with students? Think about what is realistic, acceptable, and frequently cited by young people as something they do to make themselves feel better”]. Post-implementation interviews were conducted with providers and student participants to gain a deeper understanding of their experience with the intervention, including their perception of the cultural appropriateness of materials. In supervision notes during implementation (69.6%), providers focused on contextual challenges such as the physical space for sessions (e.g., “Need to do something about the noise levels in the clinic; lots of background noise from other counselors and construction”), and its impact (e.g., “Difficult to ask kids to relax in this environment when I cannot even relax”). These contextual challenges prompted discussions about how to work with school staff to reserve dedicated space for sessions, as well as conversations about the clinical benefits of learning to use relaxation skills in situations that aren’t conventionally relaxing and are therefore more reflective of real-life settings in which these skills would be applicable.

#### Engagement

Related to student motivation to participate in the intervention, the *Engagement* code arose in 71.6% of documents, including 60% of pre-implementation meeting notes, 59.1% of implementation meeting and field notes, and 95.7% of supervision meetings during implementation. Pre-implementation conversations about engagement revolved heavily around the evidence base and focused on individual-level psychological factors, such as the suggestion to “use engagement strategies available from the literature and include in workbook. For example: instilling optimism that the program works; setting expectations about what to expect from counseling; use of motivational enhancement techniques.” In contrast, during supervision meetings held in the implementation phase, providers described barriers to engagement at the structural and contextual levels, including those related to school stressors and logistics, such as, “students aren’t even showing up to sessions because of the exam period; academic stressors take precedence.” These findings demonstrate that while the literature has identified strategies to enhance engagement, not all barriers to engagement can be anticipated and mitigated prior to implementation in a new context. The concerns raised in supervision were presented to the IWG, and possible solutions were discussed, such as being more explicit with students about the number of Step 2 sessions: “Knowing how many sessions we had left was good for adherence in Step 1. It was often requested to speed up treatment based on this knowledge […] Not knowing the treatment duration may be anxiety provoking for students and lead to dropouts.” The relationship between engagement and treatment duration informed later revisions to the treatment that aimed to reduce dropout by capping the number of sessions (37).

#### Level of care

The *Level of Care* code concerned discussions on the appropriate level of care for youth participants, including discussions on how to transition students from Step 1 to 2 and how to provide continuity between steps. The *Level of Care* code was applied in 77.3% of implementation meeting and field notes, 69.1% of pre-implementation meeting notes, and 60.9% of supervision notes. This code often highlighted deliberations about when certain practices should be given and why: the IWG team considered that “problem-solving should be kept in Step 2 and should be the feature that provides continuum between the 2 steps” and “Step 2 can be designed as a continuation of Step 1 options library.” Ultimately, the team decided to remove problem solving in Step 2 to focus on new skills deemed to be indicated for students who had not derived clinical benefit from problem solving in Step 1. Another challenge related to continuity between episodes revolved around the change in provider between Step 1 and 2. Transitioning between steps appeared to impact engagement. One provider noted that “maximum dropout happens during transition from Step 1 to 2. In typical school environments, there is one counselor. Kids also may prefer to stay with one provider and may not understand why they are being moved between steps and providers – this could interfere with engagement and contribute to difficulties building rapport.” This finding was replicated in a later pilot (37), leading the team to modify the treatment to be a single-provider model rather than having students change providers between levels of care.

#### Practice content

The *Practice Content* code captured discussions related to developing and teaching concrete and behavioral aspects of Step 2 practices. It was applied 78.4% across document types, and its application varied across the pre-implementation phase and implementation phase (i.e., 70.9% to 81.8% and 82.6%).

At pre-implementation, discussions focused more on the ‘what’ of practice delivery. For example, the team noted the need to “embed some cognitive work in the beginning of the behavioral modules to help students understand the rationale behind learning these skills […] but save cognitive restructuring for a separate module.” In contrast, during implementation, discussions more commonly focused on the ‘how’ of practice delivery. For example, during group supervision, a provider asked, “How did you introduce the idea of learning yet another skill? Find it hard to know what to say when certain things haven’t worked,” and another provider suggested using collaborative decision-making as a solution: “give students the option of practicing skills that appear to have worked for them based on the reduction in their scores or learning something else new that might help. Whatever it is, you want to be integrating the skills they learned previously.” The team also highlighted the types of problems seen in students and whether they aligned with expectations, such as when one provider stated that “in the manual, we use a specific phobia as an example, though that hasn’t come up in our sample; we should revise the manual to fit the problems that have come up (social anxiety).” These discussions resulted in proposed modifications to emphasize shared decision-making in future iterations of the protocol (37) and revise examples in the protocol to be a stronger fit with the actual problems experienced by participating students.

#### Student complexity

The *Student Complexity* code captured discussions about making Step 2 content and material appropriately complex for student participants. It was applied across 75.2% of document types and was more prominent during implementation supervision sessions (95.7%) than at pre-implementation (61.8%).

The specific topics of these discussions shifted across the pre-implementation and implementation phases. Prior to implementation, the IWG noted the importance of incorporating visuals to facilitate student comprehension and reduce burden, e.g., “Instead of handout, could have flashcards, flipchart, vicious cycle with blanks to fill in. Have something visual/graphic that is interactive; eliminate writing as much as possible.” Although the design team incorporated this recommendation by created materials that students described as visually pleasing and acceptable at the exit interview, other issues related to the content’s complexity cropped up at implementation. For example, during implementation, counselors recognized the benefits of using vignettes, noting that the students “like the scripts, it helps make concepts more understandable and concrete,” and they brought this suggestion to the IWG. In supervision, a provider described a student’s difficulty grasping content in a certain module: “Taking some time to understand the concepts. […] Provider had to explain more and go more slowly with him.” These concerns were shared with the IWG, and potential solutions were documented in meeting note discussions. Following a later pilot (37), the student-facing materials were redesigned to be consistent with the illustrated booklets used in Step 1, providing consistency between steps and meeting students’ preferences.

#### Provider complexity

The *Provider Complexity* code included discussions about how to balance complexity of the multi-problem nature of the intervention with making it simple enough to be used by non-specialist providers. This code was applied in 63.6% of the meeting and field notes during implementation. In IWG meetings, the group agreed with providers’ suggestion to add more structure: “Struggling with material: We can try and make manual more structured, which can give the beginner therapist more structure, but as they become experienced, they can have more flexibility.” The development team responded to this feedback in real time, adding suggested prompts to the manual to give providers more structure. In supervision meeting notes, usage of this code increased to 73.9%. These data highlighted themes of provider development and growing provider capacity, including a desire for more complexity within the intervention. One provider remarked in supervision, “At first, I tried to stick to the manual as much as possible. I later realized you can consolidate certain sections and adapt to the child.” This comment reflects providers’ growing confidence in their skills and ability to be more flexible in delivery, which contrasts with earlier comments about the desire for more support. From these findings, the team recognized the need to more explicitly discuss with providers the challenges associated with learning a new treatment, as well as the anticipated developmental pathway to more confident and flexible treatment delivery.

#### Clinical decision-making

Providers described a need for more support with clinical decision making, another component of a highly complex protocol. The *Clinical Decision-Making* code included discussions about how to guide providers to make decisions about which problem to target and which module to choose for that problem, as well as decisions about transitioning between modules, repeating content, and ending treatment. It was applied in 86.4% of meeting and field notes and 78.3% of supervision notes. In supervision, providers expressed concern over the lack of clarity about how to select a treatment focus: “What is the information we need to gather to make a decision about [treatment] flow after relaxation? Are we looking at scores? Progress monitoring tools? Goals? What variables do we need to look at to better understand which flow [i.e., practices] to take?” A potential solution was offered in an IWG meeting: “Need to have more structure/prompts/script in the manual for the therapist to gather information about the student’s problem. Difficult to decide what flow the student should go into if there isn’t a lot of information.” Solutions offered across meetings varied and were often discussed for months before the group decided on changes for the next research phase, including using the YTP as the primary measure informing decisions, along with emphasizing shared decision-making with the adolescent (37).

#### Monitoring

Discussions about *Monitoring* typically revolved around the pros and cons of different routine outcome monitoring measures. During implementation, the *Monitoring* code was applied in 63.6% of meeting and field notes and in 56.5% of supervision notes. The team evaluated each measure’s clinical utility while considering the need to reserve most of the session time for skills teaching. Early in implementation, a provider noted, “Progress monitoring is becoming burdensome for student […] reduce the [number of] progress monitoring tools if rich data can be captured through one or two assessments.” Once providers had sufficient piloting experience, an IWG member shared the provider consensus as “the YTP [Youth Top Problems] is most useful, takes less than 5 min. [A different measure] is difficult for the student to read, they don’t reflectively think about responses.” This feedback informed the team’s decision to retain the YTP as the primary routine outcome monitoring tool in later phases of piloting (37).

#### Student-facing materials

The *Student-Facing Materials* code was applied in 80% of pre-implementation meeting notes and 59.1% of meeting and field notes during the implementation phase. During pre-implementation, a significant amount of time was spent discussing the content of student-facing worksheets, handouts, flipbook illustrations, and routine outcome monitoring tools. The UCLA team collaborated with the Indian development team, including providers delivering Step 1 who were familiar with student preferences, to design materials in a rapid, iterative process. This approach resulted in student-facing materials that were deemed to be “Overall, more acceptable now!” prior to implementation. During implementation, comments about student materials were more related to the number of materials providers needed to manage and store, such as the fact that “Step 2 has a lot of material – able to find place for it in the clinic but need to think about where to store everything in schools; hard to find place/carry things.” The decision to use illustrated booklets rather than numerous materials in later piloting of Step 2 addressed this concern (37).

## Discussion

This study used qualitative methods to identify key challenges and considerations that arose during the development of a MH intervention for adolescents in India. Although specific aspects of this process (41, 47) and its overarching theory (36) have been described in detail elsewhere, the present study uniquely revealed ways in which diverse, cross-national teams collaboratively negotiated the terms of intervention design and reached consensus on complex decisions using a combination of local knowledge and the broader evidence base. Results support the utility of including qualitative methods in treatment development research to capture nuanced discussions from multiple perspectives and address challenges as they emerge.

Findings illustrate how priorities shifted throughout implementation and highlight the types of issues most relevant to different groups of team members. The prevalence of topics and descriptive exemplars of their content shed light on ways in which the treatment development team successfully anticipated and pre-emptively addressed potential challenges, as well as the protocol components and considerations that benefited from additional discussion during later phases of implementation. These findings have implications for the next phase of the PRIDE research project and for the future of treatment design in LMICs and HICs alike, as they illuminate intervention development issues likely to require substantial time and consideration in the design process.

As described earlier, the treatment design team pursued the *assemble* strategy for Step 2 treatment development after considering *adopt* and *adapt* approaches and deciding that existing candidate interventions did not meet the needs and preferences of the context (36). Results from the current study suggest that the collaborative Step 2 design process facilitated exchange of ideas between the local (Indian) implementation team and the UCLA intervention design laboratory, and that most major design considerations identified at the outset of the design phase remained relevant throughout, though the content of discussion varied depending on stage of implementation and discussants.

During the development and implementation process, and across document types, we found that the following codes consistently emerged: *Provider-Facing Materials, Cultural/Contextual Considerations, Engagement, Level of Care, Practice Content*, and *Student Complexity*. The consistency in their prevalence underscores their importance to the design process. This finding suggests that although treatment developers may anticipate and partially mitigate challenges in these areas at pre-implementation, on-the-ground challenges are to be expected. These difficulties may stem from many factors, including the nuanced complexities of culture that may only reveal themselves within contexts, students’ understanding of practice content once they attempt to enact it, and the ways in which providers interface with materials when using them in real-life sessions. Rather than seeing the persistent presence of these codes as a failure on the part of the design team to anticipate and address challenges prior to implementation, it may be helpful to recognize that it is expected and ultimately beneficial that these topics were discussed throughout the implementation phase and across team members.

At the same time, the downward trend in the Cultural/Contextual code frequency from pre-implementation to implementation suggests: (1) robust discussion of culture/context prior to implementation may have been effective in reducing related challenges, and (2) at the earliest stages of treatment development, the design team made satisfactory efforts to build a protocol to fit the culture and context. Similarly, student-facing materials were discussed at length in the pre-implementation phase and were raised less frequently during implementation. The UCLA design team spent a considerable amount of time prototyping student materials and eliciting feedback from the India design team in the pre-implementation phase (48). These efforts may have led to student-facing materials that, per providers’ perceptions during implementation and student exit interviews, were largely acceptable to student participants.

The frequent conversations about clinical decision making and provider complexity, as evidenced in the application of these codes, likely contributed to the IWG’s focus on monitoring, assessment, treatment architecture, and quality assurance and improvement, all of which were prominent codes in implementation meeting and field notes. In addition to revising the manual to include more guidance for providers, the team responded to providers’ desire for more support by creating and testing a one-page decision-making resource that was found to be both effective and acceptable (47), and which providers began using regularly when making decisions about treatment flow.

As stated earlier, the treatment design team desired to allow for treatment protocol flexibility and case-specific tailoring by distinguishing between clinical procedures that they determined in advance (design-time) from those that providers could select in the moment within the therapeutic context (run-time) (36). Building in a distinction between these two categories is a key feature in effective and widely implemented treatments [e.g., MAP; (30)], and enables the structured, evidence-informed system to become a useful, direct service “toolkit.” Thus, the aim was for Step 2 providers was to have enough support from the manual, training, and supervision (all products of design-time) to make run-time decisions about a specific clinical case. At the treatment development stage in the current study, however, achieving balance between design-time and run-time design was difficult. Given that this study was the initial case series of the Step 2 treatment, challenges emerged that would not arise in a more advanced stage of treatment development, when the complexity and uncertainty associated with trialing a new intervention in a brand-new context has passed.

These challenges impacted providers’ level of comfort making run-time decisions, such as how many sessions to extend a module, because they felt they lacked the clarity and autonomy to do so. The design team specified parameters regarding the types of modifications that could be made without consensus from the larger team. Modifications that could be made without approval from the IWG were minor and largely related to the interface (look and feel) of the protocol. Issues that were structural in nature and about content were brought to the IWG for discussion. Changes to these content areas were not made until a critical threshold of students and providers engaged with the materials in question. Therefore, providers had to tolerate uncertainty as the IWG waited on more data to help them come to decisions about design modifications, which limited providers’ run-time decision-making abilities. The tension of that process emerged in the current qualitative findings, as well as the provider exit interviews.

As uncomfortable as that phase of the process may have been for providers, as reflected in their comments during supervision meetings about the lack of clarity around certain design considerations, allowing the team to make data-driven decisions about protocol revisions was considered preferable to making too many *a priori* design assumptions without justifiable evidence. When revisions were deemed necessary, they were typically made to solidify key structural design considerations, often with the aim of giving providers tools to support them in making run-time decisions. For example, the decision-making resource pilot (described above) with an availability sample of local Indian counselors was launched in response to Step 2 providers’ expressed challenges with deciding between treatment flows (47). In future treatment development initiatives, there should be clear communication with study providers regarding the complexity of early implementation to set realistic expectations and buffer against loss of confidence when the challenges are a more likely a product of the phase of implementation than skillset. At the same time, providers’ potential to master and effectively deliver complex treatments should be recognized and appreciated. An dynamic intervention that grows with providers keeps them engaged and appropriately challenged while enabling them to better meet the varied and often complex needs of individuals they serve.

Building an effective, sustainable protocol for the Indian school context was the overarching goal of the Step 2 development process. The current study brings to light some of the steps taken in service of that goal: conversations across continents and cultures in pursuit of crafting an intervention to help close the treatment gap and improve adolescents’ lives. These steps, although incremental on their own, have implications for the future of MH treatment development and implementation when taken together and considered within the context of our increasingly globalized world. As described above, during early phases of treatment development and piloting, consistent communication between the remote design team and on-the-ground team members using the protocol was essential. Having the whole team in one location with access to both the evidence base and local knowledge could make this process more seamless. However, we might also see that, looking into the future of treatment design in global mental health, there will be different teams who focus the majority of their work on content vs. interface design considerations, as in the current study, especially as advances in technology make long-distance collaboration easier. Each approach has its merits, and both options increase the potential to scale up treatment innovation and reach, especially if combined. Being open to reimagining the landscape of MH in this way allows for new ideas and exchange of information and knowledge flowing both ways – not just from the West to the rest of the globe. Adopting a “glocal” perspective, which breaks down artificial barriers between global and local research, facilitates a richer, more cohesive understanding of problem areas that have largely been found to be consistent across settings while considering how cultural and structural issues impact treatment and implementation. The current study moves us in that direction by adding to the evidence base on treatments for adolescents in LMICs, highlighting critical considerations in the process of designing a treatment in collaboration with individuals from a range of cultural and experiential backgrounds, and centering feedback from stakeholders whose real-life experiences with the treatment are essential to its success.

### Limitations and future directions

The present study has many strengths, including its in-depth qualitative investigation of the multi-phasic process of building and testing an intervention using an *adopt-adapt-assemble* model with the aim of maximizing fit and scalability of an evidence-informed treatment in a low-resource setting. However, we must acknowledge its limitations. The sample size is limited, and therefore it is difficult to quantitatively examine whether differences in the types of topics raised in various document types might be attributed to background characteristics, such as training and experience, of participants. Additionally, although the coded documents were selected because they provided the most complete picture of treatment development, the complete range of discussions related to design and implementation was not captured in the current study. Relatedly, it is possible that providers may not have felt comfortable raising certain issues in supervision knowing that the meetings were documented. In future studies on intervention design, providers might be encouraged to keep field notes on their individual experiences to submit anonymously to the design team to facilitate open feedback without fear of judgment.

Another limitation concerns lack of direct student input during this design phase. Although providers reported on their experience in session and described the ways in which students engaged with content and materials, students were not approached about their experiences until they completed exit interviews at the end of treatment, giving us their perspectives at a single time point. Future studies may want to introduce a mechanism through which students can anonymously provide regular feedback to the design team throughout treatment without worrying about it impacting the therapeutic relationship with their provider. There may also be therapeutic benefit in providers using shared decision-making with students to address their concerns and explore treatment preferences. Involving youth in treatment development early in the process is another promising avenue for increasing an intervention’s fit and acceptability while simultaneously giving youth agency in designing the future of MH (49).

## Conclusion

These limitations notwithstanding, this study offers a valuable, unique contribution to the literature on youth MH treatment development and implementation in LMICs. Along with its companion papers (36, 37, 41, 47, 50), the present study addresses a gap in the literature on the process of selecting, designing, and implementing a multi-problem, modular treatment in a low-resource setting. It flags potential challenges to anticipate when designing for MHWs with varied training and experience delivering structured EBTs that are high in both complexity and utility.

Importantly, these findings highlight that despite a year of empirical fitting and context review, workforce consideration, meetings and collaborations, there were still many issues to contend with during prototyping and early testing. Looking ahead, treatment designers should expect protocol field testing to involve considerable adjusting, expectation resetting, and time needed to communicate and litigate concerns openly, regardless of how carefully the initial blueprints are laid out. Taking this approach requires patience, flexibility, and a willingness to meet providers where they are at in terms of comfort and skill when they begin learning a new protocol while simultaneously recognizing their potential to master skills and even exceed expectations if given the right support at each learning stage. The approach also asks that the treatment design team build a protocol that grows in tandem with providers rather than one that is static – perhaps an unconventional way to think about EBTs, but one that better meets the needs of a diverse MH workforce eager to be challenged.

Another key takeaway from the current study is that building a treatment using the “adopt” option of importing EBTs is nearly certain to be a poor choice for almost any context, and even “adapt” approaches are likely to have a huge design burden, simply because fitting to the final context is not easy. Lessons learned from this process can be applied not only in other LMICs, but also in HICs, where culture, context, and complexity are equally important to consider in designing MH treatments. This perspective is particularly relevant as populations in the U.S. and elsewhere become increasingly diverse and where the treatment gap, although markedly lower than in LMICs, is still a barrier to meeting the needs of adolescents experiencing MH difficulties – a problem that remains a common challenge for the global community.

## Data availability statement

The datasets presented in this article are not readily available because our approved IRB states (along with the consent forms signed by participants) that the data must not be shared with anyone outside of our research team. Requests to access the datasets should be directed to RG, resham.gellatly@bmc.org.

## Ethics statement

The studies involving human participants were reviewed and approved by Institutional Review Boards at the University of California, Los Angeles, Harvard Medical School, Sangath, and the Indian Council of Medical Research. The patients/participants provided their written informed consent to participate in this study.

## Author contributions

RG, BC, and VP contributed to the study conception and design. RG, MB, DM, KM, SM, PN, and KK contributed to the development of materials. RG and MB were performed data collection and analysis. RG wrote the first draft of the manuscript. KK contributed to the editing. All authors commented on previous versions of the manuscript, read, and approved the final manuscript.
